# Case report: chronic relapsing cryptococcal meningitis in a patient with low mannose-binding lectin and a low naïve CD4 cell count

**DOI:** 10.1186/s12879-019-4515-0

**Published:** 2019-10-15

**Authors:** Alex Wagemakers, Cornelis Willem Ang, Ferry Hagen, Joost Cornelis Johannes Bot, Marije Kristianne Bomers, Marieke Christine Visser, Karin van Dijk

**Affiliations:** 1 Department of clinical microbiology, Amsterdam UMC, Vrije Universiteit Amsterdam, Amsterdam, the Netherlands; 2 Westerdijk Fungal Biodiversity Institute, Utrecht, The Netherlands; 3 Department of Radiology, Amsterdam UMC, Vrije Universiteit Amsterdam, Amsterdam, The Netherlands; 4 Department of Internal Medicine, Amsterdam UMC, Vrije Universiteit Amsterdam, Amsterdam, The Netherlands; 5 Department of Neurology, Neurosciences Institute, Amsterdam UMC, Vrije Universiteit Amsterdam, Amsterdam, The Netherlands

**Keywords:** Cryptococcal, Meningitis, Cryptococcosis, *Cryptococcus neoformans*, Chronic

## Abstract

**Background:**

Cryptococcal meningitis is most commonly found in HIV-infected patients. In HIV-negative patients, its low incidence can lead to prolonged time to diagnosis. Detailed case reports of chronic cryptococcal meningitis are scarce, but could provide clues for earlier diagnosis in this patient category.

**Case presentation:**

A 60-year old man presented June 2015 with intermittent headaches for several months without any fever. Initial work-up showed a leukocytosis, raised CSF opening pressure and raised leukocytes and protein in the CSF. An MRI revealed leptomeningeal contrast enhancement and cerebellar oedema. While malignancy and various infectious causes were excluded, the patient had a spontaneous clinical and radiological recovery. One year later, the patient returned with complaints of headaches. Also, cerebellar oedema and leptomeningeal contrast enhancement had recurred. Eventually in March 2017, the novel cryptococcal antigen lateral flow assay (CrAg LFA) was positive on CSF, and one colony of *Cryptococcus neoformans* was cultured from CSF. The patient was treated with the standard antifungal regimen which resulted in resolution of his headaches. In retrospect, the cryptococcal antigen test was already positive on a serum sample from June 2015. Interestingly, post-treatment immunological analysis revealed both a low mannose-binding lectin (MBL) concentration and low naïve CD4 counts.

**Conclusions:**

We present a patient with cryptococcal meningitis in an HIV-negative patient with low MBL and low naïve CD4 count suffering a chronic relapsing meningo-encephalitis with relatively mild symptoms for around 2 years. In patients with an unexplained meningo-encephalitis such as this case, early performance of CrAg LFA on serum and/or CSF is an inexpensive and rapid method to reduce time-to diagnosis.

## Background

Cryptococcal meningitis is most often diagnosed in patients infected with HIV, and is considered rare in immunocompetent patients. A recent study in HIV-negative patients with cryptococcal meningitis in China showed that almost 10 % had a delayed time to treatment of > 90 days [1]. However, detailed case reports are scarce, and could provide clues for when to consider cryptococcal meningitis in apparently immunocompetent patients. Moreover, the recently introduced lateral flow assay, being very low-cost and having a superior sensitivity and specificity, could provide a key tool for diagnosis in apparently immunocompetent patients with unexplained meningitis.

## Case presentation

A 60-year old man was referred to our academic neurology outpatient clinic in June 2015. One month before he was admitted at a local hospital complaining about 3.5 months of relapsing diffuse headaches lasting several days. Only once had he developed nausea and vomiting, and he had experienced a 15-min period of diplopia. He also complained of back- and shoulder pain. The patient had not developed fever, but did describe sporadic night sweats. The patient had a blank medical history and there were no abnormalities during neurologic examination. An MRI had revealed cerebellar oedema and leptomeningeal enhancement, which resembled meningitis carcinomatosa (Additional file 1: Table S1). A lumbar puncture was performed, and CSF investigation revealed pleiocytosis (325 leukocytes/μl). Also, a raised opening pressure (25 cm H_2_O) and elevated protein (990 mg/L) were detected (Additional file 1: Table S2). Examinations for tuberculosis (IGRA, tuberculin skin test, PCR on CSF) were negative.

The patient was referred to our outpatient clinic with a differential diagnosis of either a malignancy or infection of the central nervous system. No signs of malignancy were observed on CT abdomen and thorax as well as on total body PET scan. Next, an MRI was repeated, which showed new leptomeningeal lesions with a reduction in cerebellar oedema. Also, focal contrast enhancement of L5-S1 was observed, consistent with a possible spondylodiscitis. A lumbar puncture on June 19th 2015 showed a persisting pleiocytosis of 301 cells/μl. Immunocytologic examination revealed this to be mostly polyclonal T-lymphocytes. CSF glucose was low (2.3 mmol/L). Serology was performed which excluded HIV, *Treponema pallidum* and *Borrelia* infection. Because his symptoms were relatively mild and due to a lack of diagnosis, no empirical treatment was started.

At his 3- and 9 months follow up visits, the patient was in a very good clinical condition, and MRIs (September 2015 and February 2016) showed a remarkable normalization of previously abnormal findings. Due to the spontaneous remission, no further attempts were made for a definitive diagnosis.

However, in August 2016 the patient returned to our outpatient clinic with persisting mild headaches, disorientation and dizziness as well as palpitations. The patient had no pulmonary or skin abnormalities upon physical examination. Neurologic examination did not reveal significant abnormalities, but MRI revealed new pathological contrast enhancement, mostly leptomeningeal. His headaches persisted during subsequent months. Further tests included a PET-scan which still revealed no focal FDG uptake, and an MRI which revealed a slight increase in abnormal meningeal contrast enhancement (February 2017, Fig. 1c). Because the MRI findings pointed towards a relapsing lymphoma as the cause of his symptoms, in March 2017 an extended effort was made to detect malignant cells and further exclude infectious causes.

**Fig. 1:**
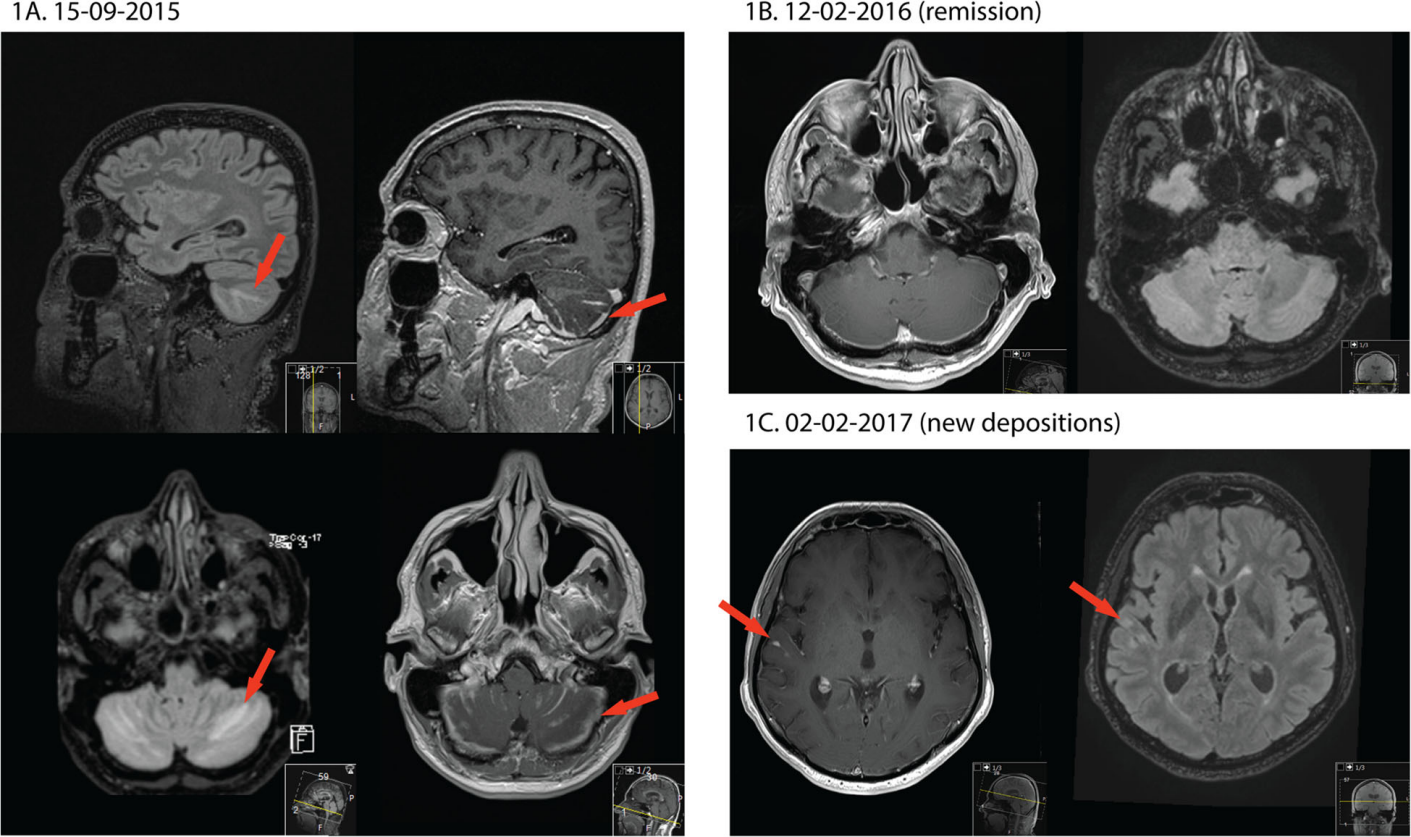
MRI FLAIR Imaging reveals cerebellar oedema. **a**: initial presentation. Sagittal (upper) and transversal images. FLAIR (left images) reveal parenchymal edema, while T1 post-contrast (right images) shows extensive leptomeningeal enhancement. **b**: normalization of MRI findings. **c**: New nodular leptomeningeal enhancement with adjacent limited parenchymal edema. Examples of radiological findings are indicated with red arrows

There was still a leukocytosis and elevated protein concentration in the CSF, but no malignant cells were observed and PCRs and antibody tests for a range of pathogens were negative. However, a new test had just been introduced in our laboratory: the cryptococcal antigen lateral flow assay (CrAg LFA; IMMY, Norman, OK, U.S.A.). This simple and inexpensive test was positive on the CSF and serum, confirming a diagnosis of cryptococcal meningitis. A fungal culture of CSF was performed, which yielded only a single colony, which by MALDI-TOF analysis (Biomérieux, Marcy-l’Étole, France) was identified to be a *Cryptococcus neoformans* (formerly *C. neoformans* var*. grubii*) [2]. Molecular characterization was performed as previously described [3], revealing the isolate to be *C. neoformans* serotype A, mating-type α, which was previously found to be the most common type among Dutch immunocompetent cryptococcosis patients [4]. The isolate was deposited in the culture collection of the Westerdijk Fungal Biodiversity Institute (accession number CBS 16101). Antifungal susceptibility testing was performed by microdilution in a reference laboratory according to EUCAST and the following minimum inhibitory concentrations (MIC) values were observed: amphotericin B 0.38 mg/l, fluconazole 4 mg/l, voriconazole 0.047 mg/l, 5-flucytosine 8 mg/l (no species-related clinical breakpoints according to EUCAST are available). Immunologic analysis of the blood showed normal leukocyte (6.9 × 10^3^/ μl), lymphocyte (1.9 × 10^3^/μl) and CD4 T-cell (760/μl) counts.

Retrospective analysis on the only available sample, serum from June 2015, resulted in a positive CrAg LFA test which uniquely demonstrates that the patient had a chronic relapsing pattern of cryptococcal meningitis for approximately 2 years. We treated the patient with an induction course of 2 weeks of L-amphotericin B (3 mg/kg 1x/d) and 5-flucytosine (25 mg/kg 4x/d), after which cryptococcal culture on CSF remained negative [5]. His symptoms mostly disappeared during the 8-week consolidation treatment with fluconazole (800 mg followed by 400 mg 1x/d) followed by 12 weeks of fluconazole maintenance therapy, after which the patient only complained about sporadic moments of very mild headaches. A year afterwards, the patient responded to an offer to evaluate the presence of non-HIV immunodeficiencies. This evaluation revealed a decreased concentration of mannose-binding lectin (MBL) of 0.47 mg/L (reference: > 0.8 mg/L). Lymphocyte typing showed a normal total lymphocyte count (2.8 × 10^6^/ml) and normal counts of total T-cells (1.95 × 10^6^/ml), CD4 cells (1.05 × 10^6^/ml) and CD8 cells (0.77 × 10^6^/ml), with a normal CD4/CD8 ratio of 1.4. Memory CD4 count was high (CD45RO+: 0.90 × 10^6^/ml), while the naïve CD4 count was relatively low (CD45RA+: 0.150 × 10^6^/ml, reference: 0.34–0.75) of which only 47% CD31+ cells. B-cell count was normal (0.27 × 10^6^/ml) as was the NK cell count (0.48 × 10^6^/ml).

## Discussion and conclusions

We present an HIV-negative patient with a chronically relapsing cryptococcal meningitis caused by *C. neoformans*, revealing a diagnostic delay of almost 2 years. Diagnosis was hampered by the relatively mild symptoms and a spontaneous recovery after extensive (negative) diagnostic work-up. In retrospect, the elevated CSF opening pressure in May 2015, combined with a leukocytosis, elevated protein and unexplained leptomeningeal abnormalities on MRI should have raised suspicion of cryptococcal meningitis. However, due to its relative low incidence in immunocompetent patients, cryptococcal meningitis is easily overlooked. In a Chinese study, only 11 out of 126 (9%) HIV-negative patients with cryptococcal meningitis had an underlying immune system disease. In the same study, while diagnostic delays of 30–90 days were common (66 out of 126 patients), only 12 out of 126 (10%) patients had a diagnostic delay > 90 days [1].

The importance of CD4 cells for immunity against cryptococci is known from the extensive experience in HIV-positive patients, while the role of MBL against cryptococcal meningitis is less described. In one study, the concentration of MBL was found to be elevated in CSF of HIV-negative patients with cryptococcal meningitis, while its plasma concentrations were normal [6]. Whether this is a result of acute phase response, or whether there is a (protective) role of MBL in the pathogenesis of non-HIV cryptococcal meningitis, and a possible susceptibility to cryptococcal infections in MBL deficient individuals, is unclear. Experimental studies should further elucidate this potential mechanism.

The mild symptoms and spontaneous remission have in our case limited the extent of diagnostics performed. Only one colony was eventually cultured, suggesting a low load, and only the recently introduced CrAg LFA has a sensitivity and specificity > 99%, offering superior sensitivity over culture, microscopy and latex agglutination [7]. In this case, we demonstrated a potential for reduction of time to diagnosis of almost 2 years by performing the antigen test on serum. Interestingly, serum antigen is usually elevated before it can be detected in CSF [8]. We advise the use of a CrAg LFA on both serum and CSF in patients with unexplained meningitis, regardless their immune status, and evaluation of the immune system in case of a positive CrAg LFA.

## Supplementary information

**Additional file 1 **: **Table S1.** Imaging findings. **Table S2.** Laboratory findings.

## Data Availability

The datasets analyzed during the current study are available from the corresponding author on reasonable request. The isolate can be obtained through the Westerdijk Fungal Biodiversity Institute under accession number CBS 16101.

## Abbreviations

CrAg: Cryptococcal antigen
CSF: Cerebrospinal fluid
FDG: Fludeoxyglucose
LFA: Lateral flow assay
MRI: Magnetic resonance imaging
PET-scan: Positron emission tomography
