# Supplementary material for: Case report: chronic relapsing cryptococcal meningitis in a patient with low mannose-binding lectin and a low naïve CD4 cell count
Source: BMC Infect Dis. 2019 Oct 15;19:846. doi: 10.1186/s12879-019-4515-0 (PMC6794769; doi:10.1186/s12879-019-4515-0)
Supplement: Supplementary file 1 — Additional file 1 : Table S1. Imaging findings. Table S2. Laboratory findings. [file 12879_2019_4515_MOESM1_ESM.docx]

**Supplemental data**

**Supplemental table 1.** Imaging findings

| Test | Date | Result |
| --- | --- | --- |
| MRI brain | 15-05-2015 (peripheral hospital) | Severe cerebellar oedema with compression of the 4^th^ ventricle and aquaduct. Diminished prepontine cistern and extensive pathological pachy- and leptomeningal enhancement in posterior groove. Possibly some intraparenchymal nodules. Most suspected of meningitis carcinomatosa. Differential diagnostic considerations: infectious causes or granulomatous disease. |
| CT thorax/abdomen | 17-06-2015 | No signs of primary or secondary tumor localization. |
| PET-CT total body | 17-06-2015 | No pathological FDG uptake, no signs of malignancy. |
| MRI brain and spinal column | 19-06-2015 | Reduced cerebellar oedema with similar meningeal and pachymeningeal nodular enhancement as was seen on 15-05. New leptomeningeal enhancement along the lateral ventricles, Virchow-Robin spaces and the pituitary stalk.  No abnormalities of the spinal cord.  Differential diagnostic considerations: granulomatous disease, inflammatory or infectious disease. |
| MRI brain  3-month follow-up | 15-09-2015 | Further reduction of cerebellar oedema, improved 4^th^ ventricle volume. Normalization of previously observed cerebellar lepto- pachy and nodular contrast enhancement. Minimal ependymal intensities at the level of the temporal horns of the ventricular system. |
| MRI brain  9-month follow-up | 12-02-2016 | Complete normalization of previously observed cerebellar oedema. Subtle ependymal ventricular enhancement at the level of the occipital horns and retrocerebellar pachymeningeal enhancement. |
| MRI brain  Return visit for persisting symptoms | 30-08-2016 | New pathological contrast enhancement, mostly leptomeningeal in both hemispheres and to a lesser extent around the medulla oblongata and pons. MRI abnormalities in time may be consistent with a primary central nervous system lymphoma. |
| PET-CT total body | 27-10-2016 | No pathological FDG uptake, no signs of malignancy. |
| MRI brain | 02-02-2017 | Slight increase in nodular leptomeningeal lesions. |
| CT thorax | 24-03-2017 | No signs of sarcoidosis |
| (MRI brain  Post-treatment initiation) | 06-06-2017 | Severe reduction of multifocal nodular leptomeningeal contrast enhancements. Only slight residual persisting contrast enhancement. Differentiation between active infection or residual scar tissue not possible |

**Supplemental table 2.** Laboratory findings

| Material | Test Date | Result |
| --- | --- | --- |
| Blood | 15-05-2015 (peripheral hospital) | CRP 6, leukocytes 10.4x10^9^/L (↑) |
| CSF | 15-05-2015 (peripheral hospital) | Chemistry: Opening pressure 25 cm H2O (↑), leukocytes 325/μl (↑) (predominantly T-cells), glucose 5.5 mmol/L, protein 990 mg/L (↑), IgG-albumin index 1.29 (↑), albumin ratio 14.8 (↑) |
| CSF | 15-05-2015 (peripheral hospital) | Pathology: no monoclonal B-cell population or abnormal T-cells, no immunocytochemical abnormalities. Most consistent with a reactive T-cell lymphocytosis. |
| CSF | 15-05-2015 (peripheral hospital | Microbiology: Ziehl Nielsen, *M. tuberculosis* PCR, mycobacterial and bacterial cultures all negative. |
| Blood | 09-06-2015 | ANA/ENA abs, rheumafactor, anti-Hu, anti-Yo, anti-Ri, anti-Tr, anti-amphi, anti-CV2, anti-ma1, anti-ma2: all negative.  Retrospectively: **Cryptococcal antigen positive (CrAg LFA, Immy; 1:10).** |
| CSF | 19-06-2015 | Leukocytes 301/μl (↑): 64% lymphocytes, 19% neutrophils, 15% monocytes, 2% other. Polyclonal, no abnormalities in immunocytology. Glucose 2.3 mmol/L (↓), protein 1627 mg/L (↑), IgM index 0.64 (↑), IgG index 1.34 (↑). |
| Blood | 29-06-2015 | *Borrelia* IgM negative, IgG equivocal, C6 peptide negative; HIV Ag/Abs negative; T. pallidum Abs negative. |
| CSF | 16-03-2017 | Gram, Indian ink and blancophor stain: negative. Leukocytes 249/μl (↑). Glucose 0.9 mmol/L (↓), protein 3222 mg/L (↑), IgG index 1.44 (↑). PCRs negative for *Borrelia burgdorferi* s.l., CMV, EBV, HSV 1/2, VZV, mycobacteria. *Borrelia* IgM and IgG negative, *Borrelia* C6 peptide negative, *Brucella* total abs negative, *Taenia* abs negative.  **Cryptococcal antigen positive (CrAg LFA, Immy; 1:160). 1 colony of *C. neoformans* cultured on Sabouraud agar.** |
| Blood | 16-03-2017 | HIV abs/ag negative.  **Cryptococcal antigen positive (CrAg LFA, Immy; 1:80).** |
| CSF  2 weeks post-treatment | 05-04-2017 | Gram and blancophor stain: negative. Leukocytes 262/μl (↑). Glucose 1.9 mmol/L (↓), protein 1853 mg/L (↑). **Cryptococcal antigen positive (CrAg LFA, Immy; 1:20).** Cultures all negative. |
